# Surveillance of zoonotic respiratory viral infections in animal farms: a model project with a focus on One Health approach–A study protocol

**DOI:** 10.3389/fpubh.2026.1854395

**Published:** 2026-09-10

**Authors:** Sudhakar Natarajan, Sriram Selvaraju, Kannan Palaniyandi, Umashankar Vetrivel, J. Nancy Hilda, Kannan Thiruvengadam, Senthil Kumar Balakrishnan, Padmapriyadarsini Chandrasekaran, Luke Elizabeth Hanna

**Affiliations:** 1 Department of Virology and Biotechnology, Indian Council of Medical Research (ICMR) – National Institute for Research in Tuberculosis (NIRT), Chetpet/Chennai, Tamil Nadu, India; 2 Virus Research and Diagnostic Lab (VRDL), Indian Council of Medical Research (ICMR) – National Institute for Research in Tuberculosis (NIRT), Tiruvallur, Tamil Nadu, India; 3 Department of Epidemiology, Indian Council of Medical Research (ICMR) – National Institute for Research in Tuberculosis (NIRT), Chetpet/Chennai, Tamil Nadu, India; 4 Department of Immunology, Indian Council of Medical Research (ICMR) – National Institute for Research in Tuberculosis (NIRT), Chetpet/Chennai, Tamil Nadu, India; 5 Epid-Statistics, Indian Council of Medical Research (ICMR) – National Institute for Research in Tuberculosis (NIRT), Chetpet/Chennai, Tamil Nadu, India; 6 Department of Clinical Research, Indian Council of Medical Research (ICMR) – National Institute for Research in Tuberculosis (NIRT), Chetpet/Chennai, Tamil Nadu, India

**Keywords:** animal farms, animal handlers, One Health, respiratory viral infection, surveillance, zoonotic viruses, pooled testing, human-animal-environment interface

## Abstract

**Background:**

The One Health approach recognizes the interconnectedness of human, animal, and environmental health in addressing the growing threat of zoonotic diseases. This study aims to establish an integrated One Health surveillance system for the early detection and monitoring of zoonotic respiratory viral infections at the human–animal–environment interface in Tiruvallur District, Tamil Nadu, India.

**Objectives:**

The study aims to establish a longitudinal surveillance system for the early detection of emerging and re-emerging zoonotic respiratory viral infections across livestock animals, animal handlers, and the farm environment interface; and ii) determine the occurrence and distribution of selected zoonotic respiratory viruses, and assess the influence of seasonal and climatic factors on their occurrence and transmission.

**Methods:**

Following sensitization of farm owners and animal handlers through the Animal Husbandry Department, 40 livestock farms from four selected blocks of Tiruvallur District, rearing cattle, buffalo, goats, sheep, swine, and poultry, will be enrolled in each surveillance round. Animal nasal swabs, respiratory samples from animal handlers, and environmental samples (air and wastewater) will be collected. Field surveillance will be carried out by a study team comprising a veterinarian and field investigators. Sixteen surveillance rounds (eight rounds annually) will be conducted over 2 years, covering two complete climatic cycles. Approximately 7,360 animal swab samples, 960 samples from animal handlers, and 960 environmental samples will be collected in each study phase. Animal samples will be screened using a pooled testing strategy followed by real-time RT-PCR for the detection of selected zoonotic respiratory viruses, while human samples will be tested individually.

**Expected outcomes:**

The study is expected to establish a robust One Health surveillance framework and generate baseline epidemiological data on zoonotic respiratory viruses circulating among livestock, animal handlers, and the farm environment in Tamil Nadu. The findings will strengthen integrated surveillance, support early warning systems, facilitate timely veterinary and public health responses, and provide evidence to inform future One Health policies and preparedness strategies for emerging zoonotic respiratory viral infections.

## 1 Introduction

The world has faced many outbreaks of infectious diseases, including the COVID-19 pandemic. India's varied geography and uneven population distribution present unique patterns for viral diseases (1). The interplay of biological, socio-cultural, ecological factors, as well as human-animal interactions, poses additional challenges to the emergence of infectious diseases. Key challenges in managing and preventing both emerging and re-emerging infectious diseases involve comprehending these factors and implementing rapid response strategies that can alleviate human suffering and prevent outbreaks (1). One Health is a collaborative, multisectoral, and transdisciplinary strategy designed to address zoonotic diseases, especially in low and lower middle-income countries (LMICs). By focusing on the connections between animals, humans, and the environment, the One Health approach could provide an effective way to deal with emerging and re-emerging zoonotic diseases (2).

Zoonotic infections, which are infections that can spread naturally between animals and humans, are increasingly posing a threat both globally and regionally. Every year millions of people and animals around the world suffer from zoonotic diseases. In Africa, diseases transmitted from animals to humans surged by 63 percent over the past decade, compared to the previous 10 years period UNEP/ILRI 2020 (3). Over 30 new human pathogens have been identified in the last three decades, including HIV, Ebola virus, West Nile virus, SARS, MERS, Influenza, Nipah virus, Hantavirus, and very recently, SARS-CoV-2, with 75 percent of these pathogens originating in animals and adapting to humans (4). Numerous animal species,–domesticated, peri-domesticated, and wild serve as reservoirs for these pathogens. Studies show that more than 1.7 million viruses are circulating in wildlife, and many of these are likely zoonotic. The highest burden of zoonotic diseases, causing widespread illness and death, is believed to be in Nigeria, Ethiopia, Tanzania, and India (5).

Animals are susceptible to some diseases just like humans, which can sometimes serve as early warning signals for potential human illnesses. For instance, birds were known to have died of West Nile virus before people in the same area became ill. Estimates show that about 60 per cent of infectious diseases and 70 per cent of emerging infections of humans are zoonotic in origin, with two-thirds originating in wildlife (6). Since most emerging and re-emerging viral pathogens come from animal reservoirs, targeted surveillance is crucial for the early detection of known and novel viral pathogens. It is estimated that 56 different zoonotic diseases cause about 2.5 billion cases of human illness and 2.7 million deaths each year (7). Livestock and companion animals also suffer disease and death following many zoonotic infections. In some cases, destruction of large-scale livestock and poultry has occurred to prevent human infections, resulting in significant economic losses. Given the public health impact of zoonotic diseases and the potential low cost of interventions, there is a compelling need to focus on developing vaccines for humans and animals (8). Vaccines have already been developed for some respiratory diseases like COVID-19, influenza, pneumonia, pertussis, and tuberculosis. However, some infectious respiratory illnesses caused by human metapneumovirus, human parainfluenza viruses, and human rhino virus still lack vaccines (9, 10).

According to a study by the International Livestock Research in India, 13 zoonotic infections have contributed to 2.4 billion cases of human disease and 2.2 million human deaths per year (11). This suggests that without timely detection, India risks facing many more pandemics in the future. There are many risk factors that contribute to the rise of emerging zoonotic diseases, and these factors are constantly changing. The significant increase in human and animal populations has raised the chances of close interaction among humans, animals, and the environment. Environmental changes, urbanization, habitat destruction, microbiological adaptation, global travel, trade in domestic and exotic animals, decline of public health systems, and the increasing number of people who are vulnerable to opportunistic infection by agents of animal origin are the major factors that drive zoonotic diseases (12).

The current project aims to set up a surveillance system for zoonotic respiratory viral infections among farm animals, animal handlers, and the animal environment. The project will identify the zoonotic respiratory viruses circulating among animals and animal handlers in the given area. Thus, the current model project envisages establishing a surveillance system for rapid identification of zoonotic respiratory viral infections among farm animals, animal handlers, and the farm environment. It will also study how climate affects the emergence, transmission, and outcome of these viral infections.

## 2 Study objectives

### 2.1 Aim 1

To establish a longitudinal surveillance system for zoonotic respiratory viral infections among farm animals, animal handlers, and the farm environment for early detection of emerging and re-emerging zoonotic respiratory viruses.

### 2.2 Aim 2

To determine the occurrence and distribution of selected zoonotic respiratory viruses among farm animals, animal handlers, and environmental samples and assess the influence of seasonal and climatic factors on their occurrence and transmission.

## 3 Proposed work plan and methodology

### 3.1 Study design: surveillance and experimental study

Study sites: Representative blocks of Tiruvallur, Poonamallee, Madhavaram, and Ponneri in Tiruvallur District of Tamil Nadu, India.

Study duration: 3-years (2024–27).

### 3.2 Study setting and study population

Tiruvallur district has an area of 3,422 sq km, with 9 Taluks and 14 blocks, and a population of 3.7 million (based on the 2011 census). The district (jilla) is divided into many subdivisions, viz. Tehsils or Taluks for administrative purposes. These subdivisions are again divided into blocks and then gram panchayats or village panchayats. We have selected four representative blocks from Tiruvallur district, which are Tiruvallur, Poonamallee, Madhavaram, and Ponneri. These four blocks were selected based on the presence of organized and unorganized animal farms. Animal farms will include cattle, buffalo, sheep, goat, swine, and poultry farms. We will also collect swab samples from the animal handlers and environmental samples (air and wastewater) from the identified animal farms.

#### 3.2.1 Eligibility criteria

##### 3.2.1.1 Eligibility criteria for animal farms and animals

**Inclusion criteria:**

A. Animal component

- The study area is located in Tiruvallur district, Tamil Nadu
- An organized or unorganized farm or a household with a minimum of 15 animals present
- Animals inhabiting cattle, buffalo, sheep, goats, swine, and poultry will be included
- Written informed consent from animal handlers for including animals in the study

B. Human component

- Individuals aged >18 years in handling or caring of animals in the selected farms/households
- Willingness to provide written informed consent

C. Environmental component

- Environmental sampling sites within the same farms/households where animals are present

**Exclusion criteria:**

A. Animal component

- Farms or households unwilling to provide written informed consent from owners/animal handlers for collection of animal samples
- Animals that are not accessible for sampling due to aggressive behavior

B. Human component

- Animal handlers who are unwilling to provide consent for collection of human samples

C. Environmental component

- Site not accessible for collection of air sample or wastewater sample

### 3.3 Sample size calculation

#### 3.3.1 Sample size calculation for animals

Sample size calculation was primarily based on the number of animal farms and zoonotic respiratory viral infections in farm animals.

There is a lack of sufficient literature and reports from Indian population on the prevalence estimates of respiratory viruses in livestock animals. Global literature from surveys of zoonotic respiratory viral infections in cattle, buffalo, sheep and goats, and swine report frequencies ranging from 10.8% to 32.4%. A study from a South-Western Polish dairy farm reported that 10.81% of animals were infected with Bovine Respiratory Syncytial Virus (BRSV) (13). Basing this report as a reference, and assuming 3% absolute precision, 95% confidence level, and an anticipated 10% loss due to field-related sample constraints, a design effect of 2 to account for clustering of animals within farms, the minimum required sample size was estimated to be 914 animals. This was rounded to 920 animals per surveillance round.

We will therefore screen 920 animals in one round and 8 rounds in a year across the selected animal farms. The surveillance will be conducted for a period of 2 years (1st phase and 2nd phase).

#### 3.3.2 Sample size calculation for animal handlers in the farms

Animal handlers will be screened in each of the selected animal farms for respiratory symptoms, and swab samples will be collected. Screening of animal handlers, and collection of swab samples will be done after obtaining their informed consent. If a respiratory viral infection is detected in animals, we will also check the animal handlers for symptoms and collect swab samples from the handlers. We propose to screen 120 animal handlers in each round from 40 animal farms for this surveillance.

#### 3.3.3 Sample size for environment samples

We will use convenience sampling to determine the sample size for the environmental samples, including animal wastewater, soil waste, and air samples from animal farms. In each round of surveillance, we will collect 3 environmental samples from 40 animal farms, collectively resulting in 120 samples. Viral RNA extraction and Real-time RT-PCR will be performed on these samples to screen for the presence of respiratory viruses.

### 3.4 Study groups on animals, animal handlers, and environment samples from animal farms in each round of surveillance

**Group A: Animals (*n* =** **920)**.

**Group B: Animal handlers (*n* =** **120)**.

**Group C: Environment (*n* =** **120)**.

The surveillance will happen every round for a period of 2 years, divided into two phases (1st phase and 2nd phase). We will conduct 8 rounds of surveillance in the 1st phase and 8 rounds in the 2nd phase. There will be a three-month break in between the two cycles for analysis of data.

Thus, 7,360 animal swab samples, 960 swab samples from animal handlers, and 960 environmental samples will be collected in phase 1 and phase 2, respectively.

**Project Implementation Plan**.

#### 3.4.1 Stratification and selection of animals from animal farms

Animal farms will be stratified based on farm size (small, medium, and large) and animal species. Farms rearing cattle and buffalo, sheep and goats, swine, and poultry will be included. Animals will be selected proportionately from each stratum to ensure representative coverage of different farm sizes and species.

According to the 20th Livestock Census conducted in the year 2017 by the Animal Husbandry Department, Tamil Nadu, the number of farm animals reported in Tiruvallur district is as follows: cattle = 282,138, buffalo = 51,239, sheep = 63,236, goats = 243,313, and poultry = 1,973. The total number of livestock reported was 641,899. The proportion of animals was cattle 44%, buffalo 7.98%, sheep 9.8%, goat 38%, and swine 0.3% (14). The proportion of animal farms used in the present surveillance study is based on data from the 20th Livestock Census.

Four blocks were selected for the surveillance study in consultation with the Animal Husbandry Department to ensure representation of major livestock-rearing areas within Tiruvallur District, considering livestock density, accessibility for repeated longitudinal surveillance, species diversity, and operational feasibility. Forty farms were selected to provide representation across cattle, buffalo, goat, sheep, swine, and poultry farm units while maintaining the logistical feasibility required for monthly surveillance over 2 years. The selected farms are intended to function as sentinel surveillance sites rather than a statistically representative sample of all farms in the district.

We will make field visits to the above-mentioned blocks in collaboration with the Animal Husbandry Department, Tiruvallur District, Tamil Nadu. Based on the number of animal farms in each of these blocks, animal farms of large, medium, and small size representative at the block level will be chosen. Random sampling will be used to stratify the animals for selection.

The number of cattle, buffalo, sheep, goats, and swine will be stratified based on the number of animals available in the large, medium, and small-sized farms. The number of animal farms planned to be covered in each block is presented in Table 1. We aim to include 40 animal farms covering 4 blocks of Tiruvallur district, Tamil Nadu, using a convenient sampling technique. Animals from small and medium farms will be randomly selected in a 1:2 ratio, while animals from large farms will be chosen in a 1:4 ratio.

**Table 1:** Proposed plan for surveillance of animal farms from 4 representative blocks of Tiruvallur district.

Blocks/Animal farms	Cattle& Buffalo	Sheep & Goat	Swine	Poultry	Total
Tiruvallur	6	6	1	3	16
Poonamallee	2	1	0	1	4
Madhavaram	2	2	0	1	5
Ponneri	6	5	1	3	15
**Total**	**16**	**14**	**2**	**8**	**40**

Bold values indicate the totals for each animal category and the overall number of animal farms included in the surveillance plan.

#### 3.4.2 Staff training

Before study initiation, standardized training programmes will be conducted separately for the field team and the laboratory team to ensure uniform implementation of study procedures.

The field team will undergo a 5-day training programme conducted by epidemiologists and veterinarians. The training will cover the objectives of the study, communication with farm owners and animal handlers, ethical conduct during field activities, safe animal handling, identification and documentation of animals with respiratory symptoms, completion of case record forms, collection of nasal swab and environmental samples, use of personal protective equipment (PPE), biosafety and biosecurity practices, specimen labeling, maintenance of the cold chain, and safe transport of specimens to the laboratory.

The laboratory team will undergo a 5-day training programme conducted by laboratory scientists and technical officers. The training will include laboratory biosafety and infection prevention practices, receipt and processing of specimens, viral RNA extraction, preparation of pooled samples, real-time RT-PCR workflow, incorporation of positive extraction controls, negative extraction controls, no-template controls, and internal amplification controls, interpretation and validation of RT-PCR results, documentation of laboratory findings, and adherence to standardized operating procedures (SOPs) and quality assurance procedures.

Refresher training sessions will be conducted periodically during the study, as required, to reinforce standardized procedures and maintain the quality and consistency of field and laboratory activities.

#### 3.4.3 Eligibility screening and interviewing

Field investigators will screen animals at farms showing respiratory symptoms, with guidance from a veterinarian. Among the 40 identified animal farms, one to two farms will be covered by the study team every day, such that all the farms under the study will be covered in one round.

Initially, the farm owners and animal handlers will be sensitized by the Animal Husbandry Department about the importance of identifying emerging zoonotic respiratory viral infections, highlighting the significance of detecting zoonotic infections for the wellbeing of the animals as well as for the prevention of disease spread to handlers and vice versa.

Each animal farm unit and animal handler will receive a unique identification number. We will obtain written informed consent in the local language before starting the symptom screening and gathering of medical history. For those who cannot read or understand, consent will be obtained from a witness, who is not part of the survey team. After obtaining consent, the participant will be interviewed to assess symptoms, and their medical history will be collected by a trained interviewer.

Accordingly, the field investigation includes standardized recording of key respiratory clinical signs by trained veterinarians using a structured case record form. These observations include the presence or absence of nasal discharge, coughing, sneezing, respiratory distress, and general clinical appearance. Animals are classified as symptomatic if one or more respiratory clinical signs are present at the time of examination and asymptomatic if none of these signs are observed (15).

Data will also be collected from symptomatic as well as random asymptomatic individuals from large, medium, and small-sized farms. We will inform the animal handlers to contact the study team if they notice any new respiratory symptoms or if any animals die.

#### 3.4.4 Screening of animals, animal handlers, and environment

##### 3.4.4.1 Screening of animals in animal farms

Nasopharyngeal swab samples will be collected from both symptomatic and asymptomatic animals, including cattle, buffalo, sheep, goats, swine, and poultry in the selected animal farms. Oropharyngeal samples will be collected from poultry. Animals will be randomly selected within each farm for the surveillance study.

If an animal tests positive for an emerging viral infection, the animal farms will be notified. They will be advised to visit the nearest veterinary hospital/clinic for further management.

##### 3.4.4.2 Screening of animal handlers

Animal handlers will be checked for respiratory symptoms. Both nasopharyngeal and oropharyngeal swab samples will be collected from those with symptoms. For those without symptoms, either nasopharyngeal or oropharyngeal swab sample will be collected.

#### 3.4.5 Data collection from animal farms

##### 3.4.5.1 Animal farm information

Supplementary Form-I will be used to collect baseline farm-level information from farm animals. It helps gather standardized data related to farm characteristics, animal population, housing conditions, and management practices that may influence disease transmission.

##### 3.4.5.2 Animal sample collection form

Supplementary Form-II will be used to systematically collect animal samples and clinical data for laboratory testing and disease monitoring. Animals will be categorized as ruminants (cattle/buffalo, goats, and sheep), swine and poultry. The following information animal information (age, gender), vaccination details, physiological status, clinical history & treatment details, and sample details will be captured in that form.

##### 3.4.5.3 Animal handlers

Supplementary Form-III will be used to collect demographic, occupational, clinical, and biological sample information from human participants involved in farm environments.

##### 3.4.5.4 Environmental sample collection form

Supplementary Form-IV will be used to systematically record environmental samples collected from farms as part of zoonotic respiratory viral disease surveillance. It ensures standardized documentation of farm conditions and environmental sample details.

#### 3.4.6 Ethical consideration

The study protocol has been approved by the Institutional Ethics Committee of ICMR-NIRT, Chennai (Ref number NIRT/2023/018), and will follow the national ethical guidelines for biomedical and health research. Informed consent will be obtained from animal handlers before including the farm animals in the surveillance study. Screening of animal handlers and collection of samples will be done after obtaining their informed written consent. Permission was accorded by the Animal Husbandry and Fisheries Department, State of Tamil Nadu, to undertake the surveillance study in animal farms in Tiruvallur district, Tamil Nadu.

#### 3.4.7 Samples to be collected from animal handlers

Animal handlers will be screened in each of the selected animal farms for respiratory symptoms, and swab samples will be collected. Nasopharyngeal sample will be collected from animal handlers who show symptoms of acute respiratory infection. Random samples from asymptomatic animal handlers will also be collected.

If a swab sample tests positive, 6 milliliters of venous blood will be collected from the animal handlers for a complete hemogram and the serum from blood will be separated and stored at −80°C in the biorepository for future use.

#### 3.4.8 Collection of environmental samples

- **Collection of air samples from animal farm units:** The collection liquid will be poured into cones and fitted with Coriolis micro air sampler (Bertin Technologies Inc., France). The airflow rate will be fixed as 200 L/min for 15 min. After that, the cone will be retrieved and fitted with the lid. The sample will be transported to the lab within 2 h in a cold chain. Environment samples, including wastewater from animal farms will be routinely collected to monitor for viral infections.

#### 3.4.9 Transport of samples to the laboratory

All swab samples from animals and animal handlers will be collected, individually labeled, and placed in viral transport medium. The air sample will be collected in the appropriate media and cone recommended by the manufacturer. A wastewater sample with a volume of 45 ml will be collected in 50 ml polypropylene tubes. The samples will be transported to the laboratory in a cold chain for testing.

### 3.5 Methods

#### 3.5.1 Aim 1

To establish a surveillance system for zoonotic respiratory viral infections among farm animals, animal handlers, and the environment for early detection of emerging and re-emerging respiratory viral infections.

Initially, we will raise awareness among animal farm owners and handlers about the importance of detecting emerging zoonotic respiratory viral infections in animals. We will emphasize how detecting zoonotic infections in animals is important for animal welfare, and for preventing disease transmission to handlers and vice versa. We will also explain the significance of the surveillance program to public and animal health and inform them that the government-funded project will cover the costs for screening of animals and humans for zoonotic respiratory infections.

We plan to include 40 animal farms across four blocks of the Tiruvallur district, Tamil Nadu. We will work in collaboration with the Animal Husbandry Department of Tamil Nadu to conduct the study. The animal farms housing cattle, buffalo, goats, sheep, swine, and poultry will be included in the study.

In the animal farms, animals showing respiratory symptoms as well as asymptomatic animals will be randomly identified, and swab samples will be collected by field Investigators under the guidance of a qualified and trained veterinarian. The study team, comprising a veterinarian, and field investigators will cover one to two farms each day. The surveillance activity in the selected farms will occur month after month for 2 years, covering the first and second phase. This way, we will gather data of zoonotic respiratory viral infections during both summer and winter seasons along with seasonal variations in infection patterns.

Each day, we plan to collect 40 swab samples from one or two animal farms. With four visits a week and 16 visits a month, the proposed field visit will lead to collection of 920 samples per round in one and half months. During each visit, we will also collect samples from animal handlers and environmental samples, including air, and wastewater.

We will request the Animal Husbandry Department to notify us promptly of any spike in zoonotic respiratory viral infections or outbreaks in the district. Similarly, we will inform them that if we notice any spike in emerging zoonotic respiratory viral infection in the farms during our study, we will immediately inform them.

Animals found symptomatic will be referred to veterinary clinics/hospitals for further management and care. Those individuals who are symptomatic or found to be positive for any of the viral infections will be referred to the nearest healthcare center for further management.

Thus, we will establish a robust surveillance system for zoonotic respiratory viral infections among farm animals, animal handlers, and the environment. This will help in the early detection of emerging and re-emerging respiratory viral infections.

Before we start the actual surveillance, we will conduct a trial run at some of the animal farms and collect a small number of samples. This will help us optimize the methods for detecting respiratory viral infections using Real-time RT-PCR. After that, we will run a small-scale trial for surveillance, collecting, *per se* 100 samples per week for a month. This will help us identify any challenges in sample collection, transportation to the lab, lab work, including viral RNA extraction, Real-time RT-PCR, interpretation of results, etc. Before starting the main study, we will identify large, medium, and small farms for inclusion. Then, we will begin the main study.

#### 3.5.2 Aim 2

To identify the zoonotic respiratory viral infections among animals and animal handlers and to study the effect of climatic conditions on the emergence, transmission, and outcome of respiratory viral infections.

##### 3.5.2.1 Biosafety and biosecurity procedures

All field and laboratory procedures will be conducted in accordance with institutional biosafety guidelines and standard operating procedures. Sample collection will be performed by trained personnel using appropriate personal protective equipment (PPE), including disposable gloves, N95 respirators, protective gowns, and eye protection. Enhanced PPE measures will be implemented during sampling from poultry and swine farms (16, 17).

Respiratory specimens will be collected into viral transport medium containing virus-inactivating reagents and transported to the laboratory under cold-chain conditions. Reusable equipment will be disinfected between farm visits using approved disinfectants, and field vehicles will undergo routine cleaning and decontamination procedures to minimize the risk of pathogen transmission between farms. Biomedical waste generated during sample collection and laboratory processing will be managed according to institutional biomedical waste management guidelines.

All molecular testing procedures, including sample processing, and nucleic acid extraction, will be handled with Class II A2 BioSafety cabinets and performed in a Biosafety Level 2 (BSL-2) laboratory with a separate pre-PCR and post-PCR rooms in Virus Research and Diagnostics Lab (VRDL) at NIRT-Tiruvallur campus.

##### 3.5.2.2 Extraction of viral RNA

Viral RNA will be extracted from swab samples collected in viral transport medium using an automated nucleic acid extraction system. Positive and negative extraction controls will be included in every nucleic acid extraction batch to monitor extraction efficiency and contamination. The VetMAX™ Xeno™ Internal Positive Control (IPC) RNA (Thermo Fisher Scientific Inc, USA) will serve as an internal positive control for the RNA purification process, and a blank viral transport medium will serve as an internal negative extraction control. Xeno IPC RNA and an internal negative control will be incorporated into the RT-PCR workflow.

**Figure 1:**
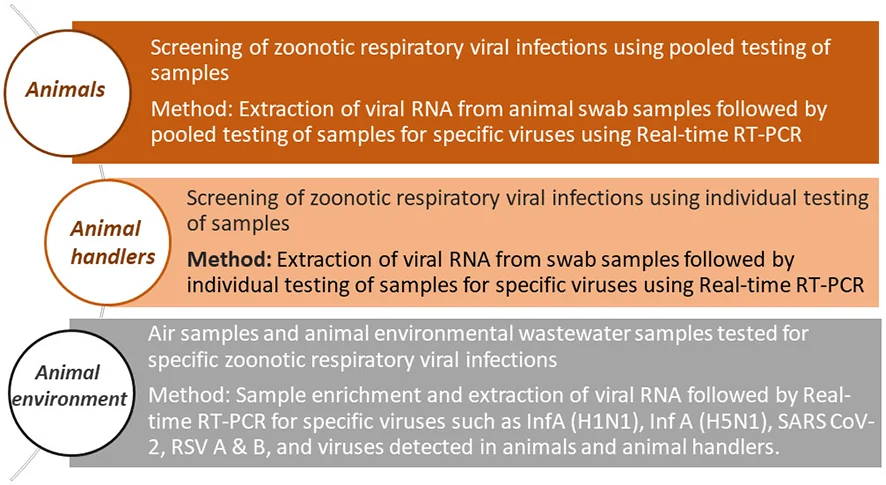
Flow diagram depicting the screening methods for detection of zoonotic respiratory viral infections in animals, animal handlers, and animal environment.

*The Schema of laboratory testing* is presented in Figure 1.

##### 3.5.2.3 Pooled testing of zoonotic respiratory infection in swab samples from farm animals

Testing by RT-PCR is highly constrained due to a shortage of reagents and limited tests that can be performed in a single run. In pooled testing, also known as group testing or pooling, a sample pool consisting of 5 viral RNA samples extracted from swab samples of animals originating from the same farm will be pooled for testing. The pools that test positive will then be tested individually (also known as pool deconvolution) (18). If a pool tests negative, likely, all individual samples in that pool will also be negative. This approach allows for testing a large number of individual samples while using the same number of tests. It can speed up the current testing system's throughput (19). The benefit of pooling is that it provides an estimate of the positivity rate in a population with fewer tests and cut down on testing time and cost.

Pooled testing will be optimized by pooling 5 viral RNA samples collected from the same farm. Livestock animals such as cattle, buffalo, sheep, and goat will be tested for species-specific viruses such as bovine coronavirus (BCoV), bovine respiratory syncytial virus (BRSV), bovine parainfluenza virus 3 (BPIV3), and avian influenza H5. All poultry samples will be pooled and tested for avian H5. Swine samples will be tested for Inf A (H1N1 pdm09) and avian influenza virus H5 infection. Pools yielding a positive RT-PCR result will undergo deconvolution, whereby all individual samples constituting the positive pool will be tested separately to identify the positive specimen(s).

We will optimize the methods for detecting zoonotic respiratory viral infections using Real-time RT-PCR with primers and probes for specific viruses. We plan to select primers and probes from WHO protocols or published papers (20, 21) and design our own primers and probes for detecting specific zoonotic viruses as needed.

Influenza A, Influenza B, and SARS CoV-2 (E gene) will be tested in all animal swab samples. Viral RNA extracted from animal swab samples will be pooled and tested for the above gene targets.

**Figure 2:**
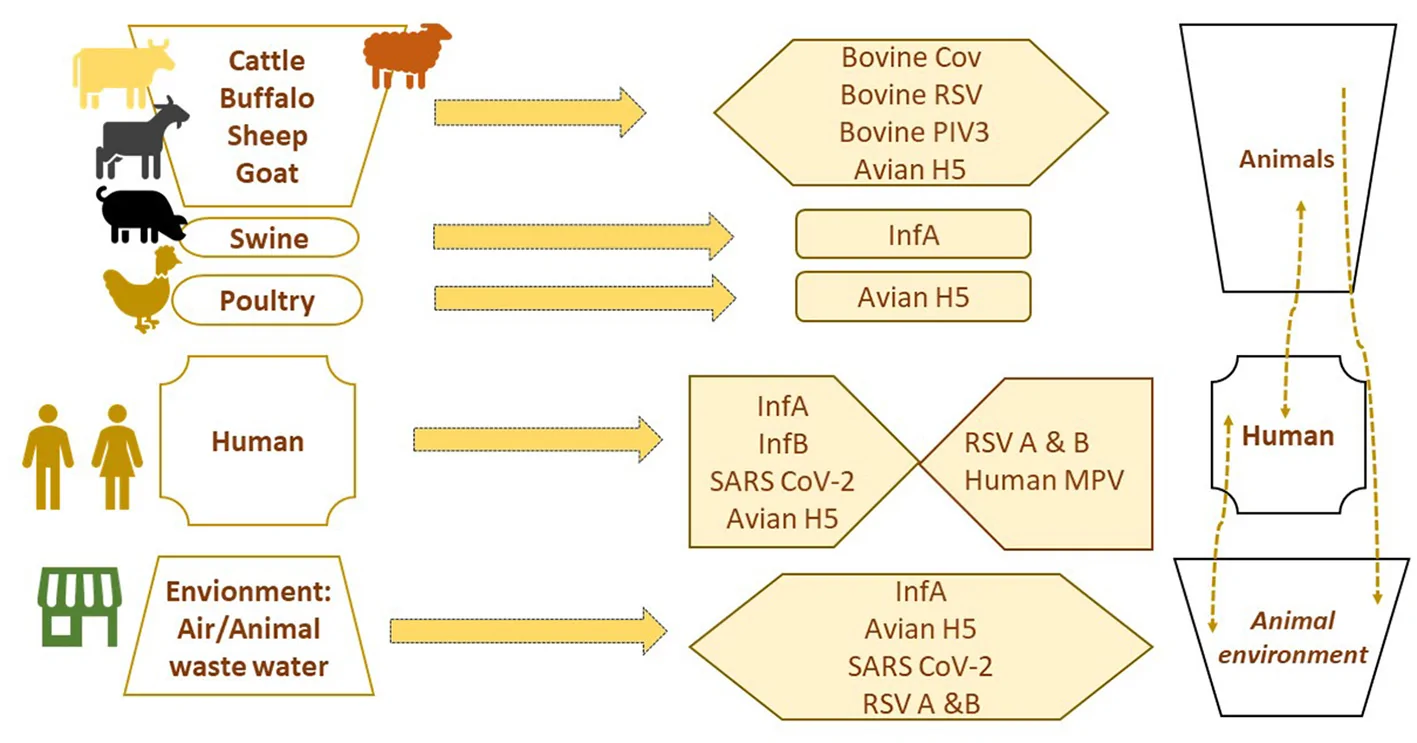
Flowchart illustrating the potential transmission of respiratory viruses at the animal-human-environment interface. Arrows indicate the possible transmission pathways of different respiratory viruses among animals, humans, and the environment.

If a sample tests positive for Influenza A infection, it will be further subtyped for Influenza A-H1N1, Influenza A-H3N2, and avian H5. If a sample tests positive for influenza B, it will be subtyped for Influenza B–Yamagata (Y) and Influenza B–Victoria (V). The laboratory testing workflow for zoonotic respiratory viral infections is presented schematically in Figure 2.

##### 3.5.2.4 Detection of respiratory viral infection in nasopharyngeal samples from animal handlers

Human samples will be tested individually for Influenza A, avian H5, Influenza B, SARS CoV-2 (E gene), respiratory syncytial virus (RSV) A & B types, human metapneumovirus (hMPV), and human parainfluenza virus 3 (PIV3). All samples that test positive for Influenza A and Influenza B will be tested for subtypes. SARS CoV-2 E gene will be tested for initial screening, and if tested positive, the N gene and ORF1ab gene of SARS CoV-2 will be tested for confirmation.

##### 3.5.2.5 Quality assurance / laboratory quality control (QC)

A positive control and a no-template control will be included in every RT-PCR assay for the individual viral target gene. A sample will be considered positive when amplification is observed with a characteristic sigmoid curve and a cycle threshold (Ct) value ≤ 38. Samples yielding Ct values >38 and ≤ 40 will be considered inconclusive and subjected to repeat testing and/or confirmatory testing before final interpretation. Samples with no amplification or Ct values >40 will be considered negative (22).

Interpretation of RT-PCR results will follow assay-specific manufacturer recommendations and validated laboratory standard operating procedures. Samples yielding indeterminate or inconclusive results will undergo repeat RNA extraction and repeat RT-PCR testing prior to final classification.

Our laboratory also participates in the external quality assurance (EQAS) program with the Virus Research and Diagnostic Lab (VRDL) network for testing viral panels, including Influenza A, Influenza B, SARS CoV-2, and RSV A and B.

##### 3.5.2.6 Real-time RT-PCR for the detection of respiratory viruses from environmental samples

Wastewater samples, and air samples from animal farms will be collected to monitor emerging viral infections.

i. The air sample from the cone will be centrifuged at 2,500 rpm for 10 min, and the pellet will be reconstituted in 1 ml viral transport medium.
ii. The wastewater sample (45 ml) will be precipitated using polyethylene glycol (PEG) 8,000 method. The wastewater will be filtered using a 0.45 μm filter to remove debris and large particulates. A solution of 10% polyethylene glycol (PEG) 8,000 (w/v) and 0.5 M NaCl will be prepared. PEG-NaCl solution will be added to the filtered sample at a ratio of 1:4 (PEG solution: sample volume). The mixture will be incubated at 4 °C for 12–24 h to allow viral particles to aggregate. Then the mixture will be centrifuged at 10,000–12,000 × g for 30–45 min at 4 °C to pellet the viruses. Then the supernatant will be carefully discarded without disturbing the pellet. The pellet will be resuspended in viral transport medium (VTM) (1–2 mL) (23, 24).

Viral RNA enrichment from wastewater samples will be done, followed by extraction of viral RNA using QIAamp Viral RNA Mini kit (Qiagen, Hilden, Germany).

Before virus concentration, each wastewater sample will be spiked with a known concentration of MS2 bacteriophage as an internal process control to monitor viral concentration, RNA extraction efficiency, and RT-PCR inhibition (25). Viral particles will be concentrated using polyethylene glycol (PEG) precipitation, followed by RNA extraction using the QIAamp Viral RNA Mini Kit (QIAGEN). Recovery efficiency will be determined by comparing the recovered MS2 genome copies measured by RT-qPCR with the known quantity initially spiked into each sample. Each batch of wastewater samples will include a negative process control (sterile water processed alongside the samples) to monitor for contamination during virus concentration and RNA extraction.

The extracted viral RNA will be tested for Bovine coronavirus (BCoV), Influenza A, SARS CoV-2, RSV A & B, and avian influenza H5 using Real-time RT-PCR. A positive control of synthetic gene fragments for the target gene and a non-template control (nuclease-free water) will be included in every RT-PCR assay. RT-PCR for MS2 RNA will be used as a process/internal control.

##### 3.5.2.7 Whole genome sequencing and genomic analysis

A representative subset of approximately 10% of RT-PCR-positive samples will be selected for whole-genome sequencing (WGS). Representative samples will be selected based on (i) a Ct value ≤ 28 to ensure adequate viral RNA for sequencing, (ii) representation across different livestock species, (iii) geographic distribution of the study sites, and (iv) surveillance rounds covering different seasons. Where multiple positive samples originate from the same pooled sample or farm, priority will be given to samples with the lowest Ct value and those contributing to epidemiological representation.

Sequencing libraries will be prepared according to the manufacturer's instructions and sequenced using an Illumina platform. Raw sequence data will undergo routine quality assessment and filtering before genome assembly. Consensus genome sequences will be generated using validated bioinformatics workflows, followed by genome annotation and lineage assignment using publicly available reference databases, including GenBank and pathogen-specific repositories such as GISAID, where applicable (26, 27).

Phylogenetic analyses will be performed using maximum-likelihood methods to determine the genetic relationships of study isolates with nationally and globally reported strains. Sequence data will be interpreted to investigate viral diversity, evolutionary relationships, and potential transmission patterns (28).

Detection of a novel viral lineage, unusual genetic clustering suggestive of local transmission, or genomic features indicating significant public health or veterinary importance will be promptly communicated to the Animal Husbandry Department and the relevant public health authorities for further investigation and appropriate response.

##### 3.5.2.8 Public health response and action

For samples obtained from animal handlers, individuals with positive results will be informed confidentially and advised to seek clinical evaluation through the local healthcare system. Relevant findings will be communicated to the District Health Authorities and the Integrated Disease Surveillance Programme (IDSP), as appropriate and in accordance with national guidelines.

Confirmed positive findings from animal samples will be communicated to the Animal Husbandry Department for further risk assessment and implementation of veterinary control measures, as appropriate under existing regulatory frameworks. Detection of zoonotic respiratory viruses with potential public health significance, identification of multiple epidemiologically linked cases within a farm, or evidence of transmission involving animals, humans, and/or environmental samples will prompt an enhanced investigation and consultation with public health and veterinary authorities (29).

If an unexpected high-pathogenicity strain (e.g., highly pathogenic avian influenza [HPAI] H5) is detected or suspected during laboratory testing, the finding will be communicated immediately to the Animal Husbandry Department and the appropriate public health authorities through established institutional guidelines. Aliquots of the specimen will be referred under appropriate biosafety and cold-chain conditions to the ICMR-National Institute of Virology (NIV), Pune, or the ICAR-National Institute of High Security Animal Diseases (NIHSAD), Bhopal, as appropriate, for confirmatory testing and further characterization. Subsequent public health and veterinary response measures will be undertaken in accordance with national guidelines and the recommendations of the competent authorities.

### 3.6 Data collection

All data collection will be done physically using structured data collection farms. The data collection farms are provided as Supplementary Table (From I to Form IV). The data captured in physical form from the field will be transferred to redcap database. Each animal farm included in the study and each animal handler participating in the study will be given a unique participant ID. The field and lab data will have individual data entry systems, which will be linked to the REDCap system.

The climate data including temperature (minimum and maximum), relative humidity, and rainfall for the study area, will be obtained from the Indian Meteorological Department, Chennai. Meteorological data, including ambient temperature (°C), relative humidity (%), and rainfall (mm), will be obtained from the India Meteorological Department (IMD) corresponding to the study area for each surveillance round. Where available, block-level or the nearest IMD weather station data will be used; otherwise, district-level meteorological data will be utilized. In addition, surveillance rounds will be categorized into seasonal periods (summer, southwest monsoon, northeast monsoon, and winter) to evaluate seasonal variation in viral circulation. The electronic data will be securely maintained using locked storage and password protection. Only study staff directly involved in data management will have access, and necessary backups will be taken on an external hard disk.

### 3.7 Data analysis plan

All study variables will be summarized using appropriate descriptive statistics. Categorical variables will be presented as frequencies and proportions, while continuous variables will be summarized using means and standard deviations or medians and interquartile ranges, as appropriate. Bivariate analyses, such as Spearman's correlation will be performed to assess association between variables and viral infections. Chi square test or Fisher's exact test will be used categorical variables, and student *t*-test or one-way analysis of variance (ANOVA) or their non-parametric equivalents used for continuous variables.

The time, place, and person analysis will be conducted to describe the epidemiological distribution of infections. The factors related to the infection among the study population will be identified using appropriate regression methods, and models will be developed to predict the future occurrence. The transmission pattern, seasonality, and incidence proportion and incidence density will be calculated. Spatial analyses will be performed to evaluate the geographical distribution of infections and their relationship with environmental surveillance findings. In addition, climatic variables will be incorporated into statistical analyses as continuous variables.

Because the surveillance data have a hierarchical structure, with animals nested within farms and farms nested within study blocks, multivariable analyses will account for clustering using generalized linear mixed-effects models (GLMMs) and/or Generalized Estimating Equations (GEE), as appropriate. Farm and study block will be incorporated as random effects or clustering variables to obtain robust estimates of associations between risk factors and viral infections while accounting for within-farm correlation. Appropriate regression models will be developed to identify factors associated with infection and to predict the occurrence of zoonotic respiratory viral infections. Associations between climatic factors and viral detection will be assessed using multivariable mixed-effects regression models while accounting for clustering of animals within farms and farms within study blocks.

The prevalence of co-infections will be estimated and compared across animal species, surveillance rounds, and seasonal periods using appropriate statistical methods.

All statistical analyses will be performed using standard statistical software, and a and a two-sided *p*-value of < 0.05 will be considered statistically significant.

### 3.8 Expected outcomes

This study is designed to establish a robust One Health surveillance framework for monitoring zoonotic respiratory viral infections at the human–animal–environment interface. The surveillance system is expected to generate evidence on the occurrence and distribution of selected respiratory viruses among livestock, animal handlers, and environmental samples across different seasons.

The study may identify circulating zoonotic respiratory viruses, characterize their temporal and spatial distribution, and evaluate potential associations with climatic and environmental factors. Whole-genome sequencing of representative positive samples, where available, is expected to provide insights into viral genetic diversity and evolutionary relationships.

Regardless of whether viral prevalence is high, low, or absent in specific species or surveillance periods, the study will provide valuable baseline epidemiological data to strengthen future One Health surveillance, inform public health and veterinary preparedness, and support evidence-based policies for the prevention and control of emerging zoonotic respiratory viral infections. The surveillance framework developed through this project is intended to serve as a model for integrated zoonotic disease surveillance in similar settings.

## 4 Discussion

The proposed investigation aims to fill a major gap in the detection and monitoring of zoonotic respiratory viral infections at the interface of humans, animals, and the environment. Zoonotic diseases, especially those caused by respiratory viruses, have consistently demonstrated their potential to cause outbreaks, which have considerable public health and economic implications. In this framework, the implementation of a One Health paradigm within a district-level surveillance initiative presents both innovative and practical benefits.

This initiative is robust because it is set up as a long-term surveillance system that includes various livestock species, such as cattle, buffalo, goats, sheep, swine, and poultry, along with the animal handlers and the surrounding environment. This model allows for the simultaneous detection of viral pathogens, reducing the chances of missing transmission events. By pooled testing methods and Real-time RT-PCR, the protocol ensures high sensitivity and cost-effectiveness, which are essential in resource-limited settings.

The current research aims to screen and assess 920 animals, 120 animal handlers, and 120 environmental samples per round for a period of 2 years (Phase 1 and Phase 2). Animals will be monitored regularly for clinical symptoms; as respiratory viral infections in animals show no symptoms, hence both symptomatic and asymptomatic animals will be included. The investigation will occur in two phases so as to obtain longitudinal data for a period of 2 years, enabling the assessment of temporal trends and seasonal variation.

Bovine coronavirus (BCoV), bovine respiratory syncytial virus (BRSV), and bovine parainfluenza virus type 3 (BPIV-3) are significant causes of the bovine respiratory disease complex (BRDC) and neonatal enteric disease. The prevalence and clinical manifestations of these viruses depend largely on factors like animal age, herd management practices, seasonal changes, and the presence of co-infections (30). Evidence from global and regional sources consistently indicates that these viruses frequently co-circulate and are often associated with bacterial pathogens and other viral agents. This combination can increase in disease severity and contributing to production losses (31).

Although SARS-CoV-2 mainly affected humans, the COVID-19 pandemic highlighted its ability to infect a wide range of animal species including mink, deer, pets, and zoo animals. Outbreaks on mink farms demonstrated that the virus can be transmitted between humans and mink, indicating that animal occupational workers might contract infections from animals. This also suggests that these animals could act as reservoirs, possibly spreading the virus back to humans (32, 33). Reviews on SARS-CoV-2 in animals stress the importance of monitoring those exposed to animals, such as farm workers, veterinarians, and abattoir workers, within the One Health framework. These animal hots can act as temporary reservoirs, potentially influencing viral evolution and transmission dynamics (34, 35).

Importantly, the study design includes selected farms across four representative blocks in Tiruvallur District, Tamil Nadu. This allows for tracking changes over time and seasonal variations in respiratory infections. This is crucial in tropical regions where factors like temperature, rainfall, and humidity can greatly influence how long the virus survives, how it spreads, and how vulnerable hosts are. The structured monthly sampling carried out over 2 years will help create solid baseline data. This data can be valuable for developing models to predict the outbreak risks.

Occupational serosurveys show that poultry and swine workers have a higher rate of antibodies against animal influenza viruses than the general public. However, the overall seroprevalence can vary greatly depending on the context, subtype, and laboratory methods (36). For example, studies in India and other regions have found low to moderate H9 or H5 seropositivity among poultry workers, often in the single-digit percent range across multiple studies. In contrast, focused outbreak investigations or groups with high exposure show higher seroprevalence (37, 38). These findings indicate that animal handlers are important for early detection of zoonotic spillover events.

There is consistent evidence that exposure at the human-animal interface results in higher rates of past or recent infections with zoonotic respiratory viruses compared to the general population. However, the specific prevalence rates vary widely based on virus type, geographic area, season, and research methods (36). Two main patterns emerge from the research: (1) point-prevalence identified through molecular techniques, like Real-time RT-PCR and viral detection, is usually low because viral shedding is often temporary; and (2) seroprevalence, which measures antibodies in occupational groups, is usually much higher, reflecting cumulative exposure over time (39, 40).

We acknowledge that the sampling strategy may limit the generalizability of prevalence estimates to all farms within Tiruvallur District. The selected farms serve as sentinel surveillance sites and were not chosen through probability-based sampling. Consequently, prevalence estimates generated through this surveillance system may not be fully representative of all livestock farms within Tiruvallur District. The findings should therefore be interpreted primarily in the context of pathogen detection, temporal trends, and early warning surveillance rather than district-wide prevalence estimation.

It is important to acknowledge the operational challenges associated with the study. Keeping farm owners engaged and working together over 2 years will likely need continuous communication, trust building, and involvement of active stakeholders. In parallel, maintaining the ability of the laboratory to perform high-throughput testing of samples with adequate manpower through the funding support is essential. Despite these challenges, the well-organized design, involvement of stakeholders, and integrated surveillance across animal, human, and environmental interface make this initiative a notable example of applying the One Health approach at a community level.

**Figure 3:**
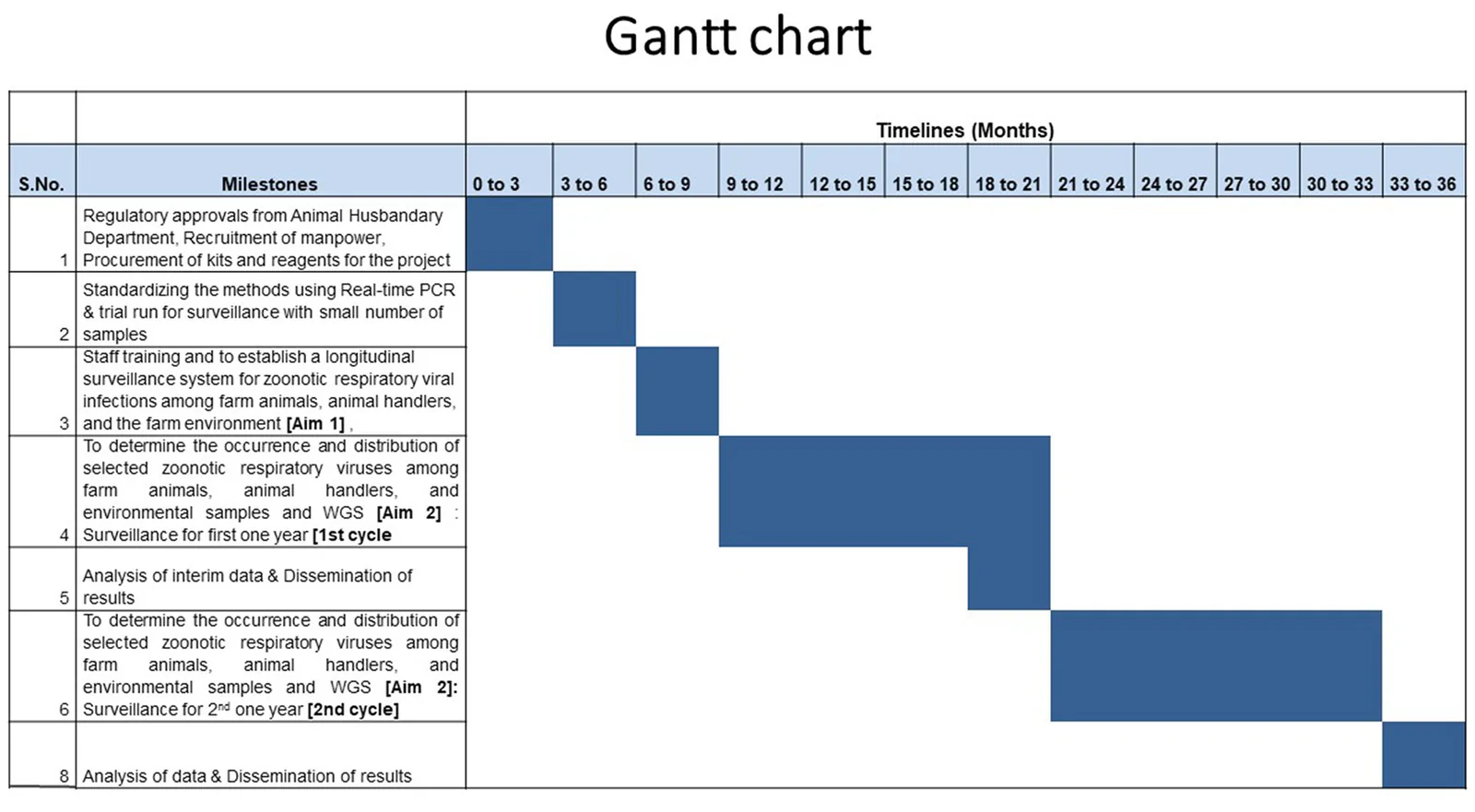
Gantt chart showing eight milestones for a project on zoonotic respiratory virus surveillance, with tasks spanning regulatory approvals, PCR method standardization, staff training, surveillance cycles, data analysis, and result dissemination mapped across 36 months.

Thus, the field-based strategies and laboratory framework in the present study have the potential to be adapted and implemented to other districts and states as a model for integrated zoonotic respiratory disease surveillance. We have included a detailed Gantt chart (Figure 3 illustrating the overall study timeline).

In conclusion, the proposed surveillance protocol will serve as a model for the early detection and response to zoonotic respiratory viral infections. By generating district level data on circulating viral pathogens, seasonal trends, and inter-species transmission dynamics, the study will contribute to the development of public health strategies. To sum up, this project has the potential to lessen the public health burden and reduce the economic impact of respiratory viral diseases in communities that rely on livestock.
