# Supplementary material for: Surveillance of zoonotic respiratory viral infections in animal farms: a model project with a focus on One Health approach–A study protocol
Source: Front Public Health. 2026 Sep 10;14:1854395. doi: 10.3389/fpubh.2026.1854395 (PMC13601349; doi:10.3389/fpubh.2026.1854395)
Supplement: Supplementary file 1 [file Data_Sheet_1.pdf]

Surveillance of Zoonotic Respiratory Viral Diseases in Farm Animals

(Farm Information) Form - I

Date : ___________

Farm Details:

Farm ID : ___________

Visit Code : ___________

1. Farm Name :
2. Farm owner name :
3. Owner mobile No :
4. Farm Address :
5. Type of farm : Organized / Semi-organized / Backyard / Mixed Farming
6. Farm population :

Livestock	Nos	Livestock	Nos
Cow (C)		Goat (G)	
Buffalo (B)		Pig (P)	
Sheep (S)		Avian (A)	

7. Nature of housing : Loose housing / Free range / Conventional barns
8. Type of roofing : Lean to roof / Gable / Monitor
9. Water source for the farm : Ground water / From Natural bodies
10. Nature of feeding : Free grazing / In house feeding
11. Method of animal identification :
12. Details of the nearest Veterinary Dispensary :

Name & Signature of Staff who filled the Questionnaire : --------------------------------

----

Signature of Project Veterinarian : ____________________

Signature of PI/Co-PI : ____________________

Surveillance of Zoonotic Respiratory Viral Diseases in Farm Animals

(Animal Sample Form) – Form - II

Farm ID :_______________ **Collection Date:**

Animal ID : ZRVI / ____ / _______ **Sample Source: C / B / S / G / P / A**

Sample ID :________________________ **Visit Code : _______________**

1. **Breed** : 1a. Avian: Broiler / Layer
2. **Age of Animal / Flock :**
3. **Nature of Breeding** : AI / Bull / Ram / Buck / Rooster
4. **Vaccination details** :

Avian Vaccines	Sheep & Goat Vaccines	Cattle Vaccines	Swine Vaccines
Avian influenza ☐	ET ☐	FMD ☐	Classical Swine Fever ☐
ND ☐	Bluetongue ☐	LSD ☐	
Marek's disease ☐	Sheep pox ☐	Brucella abortus ☐	
ILT ☐	Goat pox ☐	Anthrax ☐	
IBD ☐	PPR ☐	HS & BQ ☐	
IB ☐		Johnes's disease ☐	
Fowl Pox ☐			
**Other Vaccines**	**Other Vaccines**	**Other Vaccines**	**Other Vaccines**

5. **Physiological status** : Lactation / Pregnancy / Dry / Others_______________
6. **Details of recent treatment history(if any):**

Clinical Signs	Duration of illness

7. **Sample Details:**

Type of Sample	***Sample Storage ID*	Remarks
**Nasal** ☐ **Oropharyngeal** ☐<br>**Others**______________________________		

Signature of Project Veterinarian: ____________________

Surveillance of Zoonotic Respiratory Viral Diseases in Farm Animals

(Human Sample Form) – Form - III

Farm ID :_______________ **Collection Date:**

Human ID : ZRVI / ____ / _______ **Sample Source: Human (H)**

Visit Code: ____________

1. **Name of the Person** :
2. **Father's Name** :
3. **Gender** : Male / Female / Others
4. **Age** :
5. **Marital Status** : Married / Single
6. **No of Children** :
7. **Total Experience / Contact No** : /
8. **No of hrs spent in farm/day** :
9. **Family Details** :
   a) **Family Status** : Nuclear / Joint
   b) **Residing with family** : Yes / No
10. **House details** :
    a) **Ventilation details** :
    b) **No of windows** :
11. **History of Illness** :

a) Details of Comorbidities :	b) Signs of respiratory issues:
**c) Signs of other diseases :**	**d) Vaccination (if any):**

12. **Sample details**

Type of Sample	*Sample Storage ID*	Remarks
Blood ☐ Nasal Swab ☐<br>Oral Swab ☐ Others ☐		

Signature of Lab Technician / Phlebotomist : ________________________

Surveillance of Zoonotic Respiratory Viral Diseases in Farm Animals

(Environmental Sample Form) – Form - IV

Sample ID : ________________________ **Date :**___________

Farm Details: **Farm ID :** ___________

Visit Code : ___________

1. Farm Name :
2. Weather conditions : Summer / Rainy / Winter
3. Nature of sample collected :

	Source	Number of samples
**Site 1** (Animal and its surroundings)	☐ Feed trough<br>☐ Water<br>☐ Sewage sample	
**Site 2** (Trespass area and farm premises)	☐ Air	
